# Effect of Selenium on Alleviating Oxidative Stress Caused by a Water Deficit in Cucumber Roots

**DOI:** 10.3390/plants8070217

**Published:** 2019-07-11

**Authors:** Weronika Jóźwiak, Barbara Politycka

**Affiliations:** Department of Plant Physiology, Poznań University of Life Sciences, Poznań 60-637, Poland

**Keywords:** selenium, water deficit, *Cucumis sativus* L., roots, oxidative stress, antioxidant enzymes

## Abstract

The aim of the study was to evaluate the antioxidant activity of selenium in the roots of *Cucumis sativus* L. seedlings pre-treated with selenium (Se) in the form of sodium selenite at concentrations of 1, 5, and 10 µM, and then subjected to a water deficit (WD). It has been hypothesized that Se, in low concentrations, alleviates an oxidative stress caused by a WD in the cucumber roots. A WD was introduced by the surface dehydration of roots. The aim of the research was to compare the changes accompanying oxidative stress in plants growing in the presence of Se and in its absence. The study concerns the generation of reactive oxygen species (ROS)—superoxide anions (O_2_^•−^), hydrogen peroxide (H_2_O_2_), and hydroxyl radicals (^•^OH)—as well the activities of the antioxidant enzymes lowering the ROS level—ascorbate peroxidase (APX), peroxidase (POX), catalase (CAT), and superoxide dismutase (SOD). A WD caused oxidative stress, i.e., the enhanced generation of ROS. Selenium at the concentrations of 1 and 5 μM increased the tolerance of cucumber seedlings to oxidative stress caused by a WD by increasing the activities of the antioxidant enzymes, and it also limited the damage of plasma membranes as a result of the inhibition of lipid peroxidation.

## 1. Introduction

Plant metabolism is often disrupted by a number of adverse environmental factors; therefore, it is important to conduct research towards the increasing ability of plants to survive in these unfavourable conditions. Drought is one of the main factors limiting crop yielding. About 45% of the world crop area is exposed to continuous or frequent drought stress, and over 40% of world-wide yields, especially vegetables, are obtained on artificially irrigated soils [1].

High water requirements are one of the reasons for restricted cucumber cultivation. This species is extremely sensitive to a water deficit (WD) [2,3], which is associated with a poorly developed and shallow root system, the production of a large mass of above-ground parts, and a high water content in fruits. Low soil moisture reduces the size and quality of a cucumber yield [4].

Various biotic and abiotic stress factors, including a WD, induce, in plants, oxidative stress, which is one of the earliest universal responses of cells revealed by a violent increase of the intracellular level of reactive oxygen species (ROS) in form of hydrogen peroxide (H_2_O_2_), superoxide anions (O_2_^•−^), and hydroxyl radicals (^•^OH) [5,6]. For the proper course of metabolic processes, it is necessary to maintain a balance between ROS generation and scavenging. Oxidative stress relies on disturbing the balance between the formation and degradation of ROS. However, when the amount of ROS exceeds the efficiency of antioxidant systems, free radical changes may occur in the cells that damage cellular components, including the oxidation of thiol groups of proteins, lipid peroxidation, and the disruption of DNA strands.

Plants have developed various defensive mechanisms that allow for ROS removal and, thus, minimize and prevent progressive damage to cellular components. The antioxidant system includes the action of small-molecule antioxidants and the induction of antioxidant enzymes.

Selenium is an essential element in animal cells and the human body, but its importance for plants is still the object of research. This element, occurring in a narrow range of concentrations, may have a positive effect on plants. In higher concentrations, however, it is toxic [7,8]. The beneficial antioxidant activity of Se has been observed under stress conditions when increased amounts of ROS are generated. The enzymes of the antioxidant system that protect cells by eliminating ROS include catalase (CAT), superoxide dismutase (SOD), and numerous peroxidases (POXs), among which ascorbate peroxidase (APX) is included. The mechanism of the antioxidant activity of Se in plants has not yet been fully elucidated. To date, it has been shown that CAT, SOD, and POX activities are induced by Se [9]. In addition to their harmful effects, ROS have the role of mediating signal transmission in the defence and acclimatization responses of cells to stress factors [10], the effects of which are shown with lower concentrations of ROS. Therefore, the regulation of certain elements of the antioxidant system by Se may affect the reduction of ROS levels and, in this way, change their role from harmful to beneficial.

Plant organisms are exposed to various stress factors that can interact or follow one another. The combined action of stress factors can modify response patterns. Studies on the interactions of unfavourable factors on plants have proven that there is a so-called phenomenon of cross-reactions that results in the increasing resistance or susceptibility to a stress factor as a result of an earlier action of another factor. Plant response depends on the intensity of stress factors, the sensitivity of species to these stress factors, the duration of exposure, and the mode of action. Protective effects are usually observed as the sequential or simultaneous actions of stress factors, especially when one of them occurs at a low, sub-lethal level [11]. Numerous studies have shown that Se, by affecting the activity of the antioxidant system, can counteract the negative effects of various stress factors, including a WD [9]. However, most of these studies concern the response of the above-ground parts of the plant. In this work, the roots are the object of the research. Roots, in addition to their structural function, play a key role in the survival of plants under unfavourable conditions. They are the first to be exposed to stress factors occurring in the soil, including a WD, and trigger a series of physiological, morphological, and molecular responses for the whole plant. The initial stress signal is further passed to other organs of the plant, a passing which is extremely important for inducing a specific response, i.e., tolerance or susceptibility to a given stress factor [12].

The aim of this study was to assess the antioxidant activity of Se in the roots of cucumber seedlings under WD conditions. The objective of the research was to compare the metabolic changes accompanying oxidative stress in plants growing in the presence of Se or in its absence. These changes relate to the generation of ROS and the activities of the antioxidant enzymes reducing their level. It has been hypothesized that Se, at low concentrations, relieves the intensity and effects of oxidative stress caused by a WD in the roots of cucumber seedlings. To verify the above mentioned hypothesis in the roots of cucumber seedlings grown in nutrient solution with Se and then subjected to a WD, changes in both ROS levels, such as superoxide anions (O_2_^•−^), hydrogen peroxide (H_2_O_2_), and hydroxyl radicals (^•^OH), in the activities of APX, POX, CAT, and SOD were monitored. What is more, the level of lipid peroxidation product—malondialdehyde (MDA)—and the degree of cell membrane damage were observed.

## 2. Results

### 2.1. Effect of Se on the ROS Level

#### 2.1.1. Superoxide Anion (O_2_^•−^)

**Figure 1:**
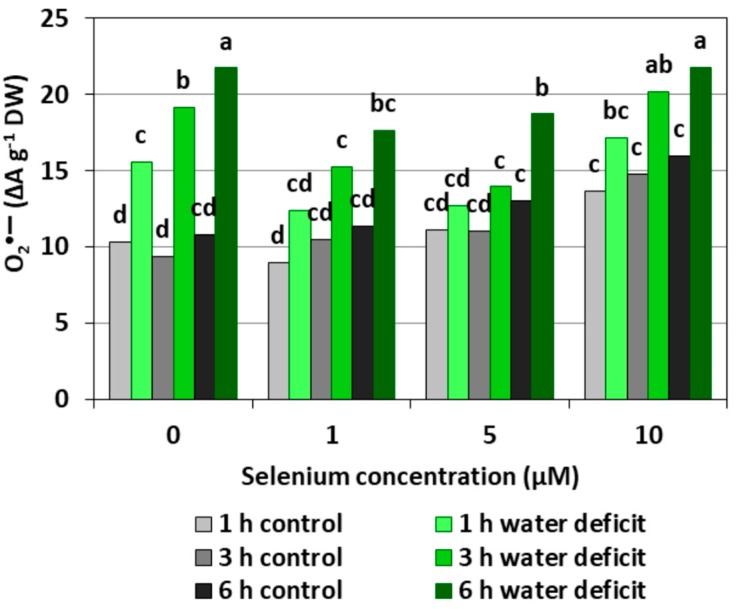
The superoxide anion radical (O_2_^•−^) level in the roots of cucumber seedlings pre-treated with selenium (Se) and subjected to a water deficit (WD). Bars marked with the same letters do not differ significantly at *p* < 0.05.

Figure 1 shows the level of O_2_^•−^ in the roots of cucumber seedlings. It was found that the pre-treatment of cucumber seedlings with Se at different concentrations caused a significant increase in the generation of O_2_^•−^ only at the highest Se concentration (10 µM), compared to the control that did not have Se. The introduction of a WD contributed to a significant increase in the O_2_^•−^ level compared to the roots of control seedlings, and the longer the WD lasted, the stronger the generation of O_2_^•−^ was. In the roots of seedlings subjected to a six hour WD, a twofold higher level of O_2_^•−^ was observed than in the roots untreated with Se and not subjected to a WD. Similarly, a strong generation of O_2_^•−^ was found in roots pre-treated with 10 μM of Se and subjected to a WD. Conversely, the generation of O_2_^•−^ in the roots of cucumber seedlings pre-treated with 1 μM of Se and then subjected to a WD for three and six hours, as well as in those pre-treated with 5 μM Se and subjected to a WD for one and three hours, was significantly weaker compared to that of the WD alone.

#### 2.1.2. Hydrogen Peroxide (H_2_O_2_)

**Figure 2:**
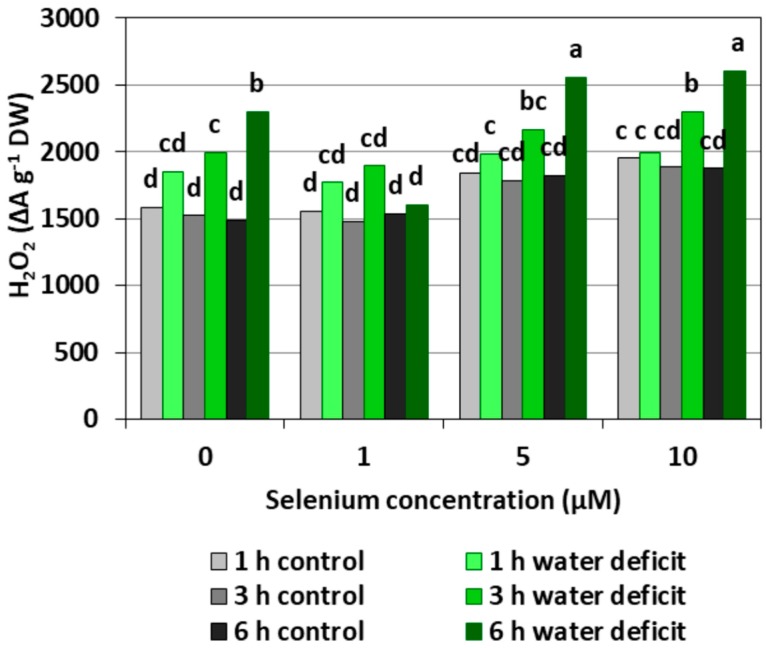
The hydrogen peroxide (H_2_O_2_) level in the roots of cucumber seedlings pre-treated with Se and subjected to a WD. Bars marked with the same letters do not differ significantly at *p* < 0.05.

Selenium pre-treatment did not cause significant changes in H_2_O_2_ generation as compared to the control without Se (Figure 2). However, there was a noticeable tendency to increase the H_2_O_2_ level with the increasing concentration of Se. The roots of cucumber seedlings grown in the nutrient solution without Se and then subjected to a WD for three and six hours showed a significant increase in the generation of this molecule compared to roots not subjected to a WD, which was not noted in the case of a one hour WD. A WD did not cause a significant increase in the H_2_O_2_ level of the roots of seedlings growing at 1 μM Se when the production of this molecule was comparable to its level in roots growing in the nutrient solution with 1 μM Se and not subjected to a WD. Selenium at concentrations of 5 and 10 μM caused a slight increase in the generation of H_2_O_2_ in roots subjected to a WD, respectively, in the sixth hour and in the third and sixth hour. The strongest generation of H_2_O_2_ was found in the roots of cucumber seedlings pre-treated with Se at concentrations of 5 and 10 µM Se and then subjected to a six hour WD. The H_2_O_2_ level was, on average, 40% higher compared to the Se treated roots without a WD.

#### 2.1.3. Hydroxyl Radical (^•^OH)

**Figure 3:**
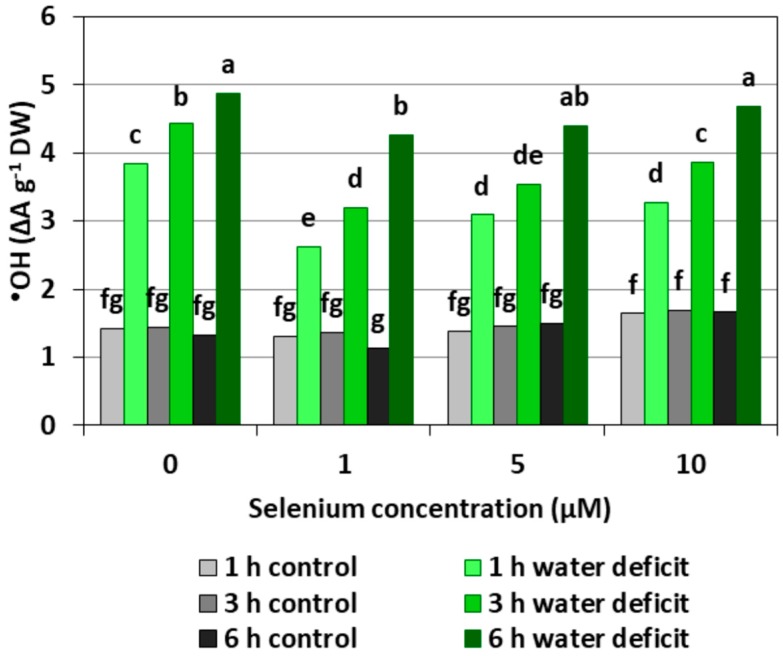
The hydroxyl radical (^•^OH) level in the roots of cucumber seedlings pre-treated with Se and subjected to a WD. Bars marked with the same letters do not differ significantly at *p* < 0.05.

The pre-treatment with Se did not cause a significant increase in the level of ^•^OH in the roots of cucumber seedlings (Figure 3). The introduction of a WD caused the strong generation of this radical, which increased with the longer action time of the stress factor. The generation of ^•^OH in the roots of seedlings with a WD was, on average, 2.4-fold higher compared to the control roots without a WD. In the roots of Se pre-treated seedlings, a weaker effect of the WD on the generation of ^•^OH was observed, with the exception of roots treated with 5 and 10 μM of Se and subjected to a six hour WD.

### 2.2. Effect of Se on the Activities of Antioxidant Enzymes

#### 2.2.1. APX Activity

**Figure 4:**
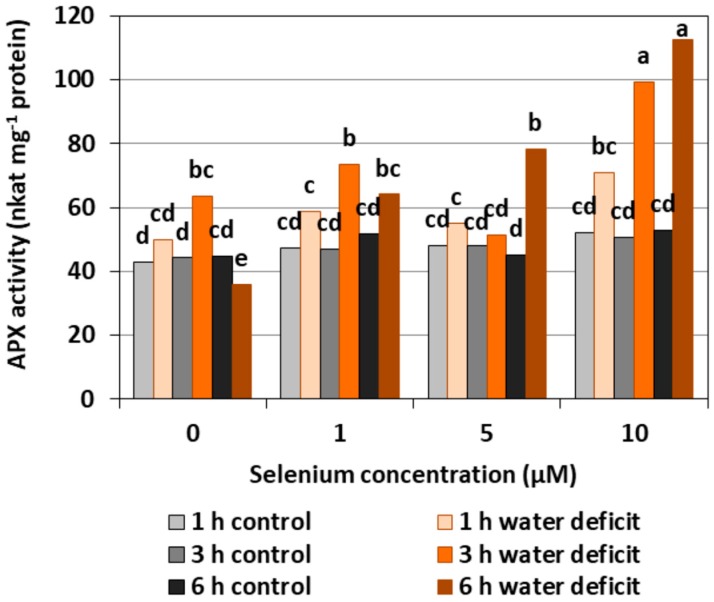
The ascorbate peroxidase (APX) activity in roots of cucumber seedlings pre-treated with Se and subjected to a WD. Bars marked with the same letters do not differ significantly at *p* < 0.05.

The pre-treatment with Se of the roots of cucumber seedlings did not affect APX activity (Figure 4). In the roots of Se untreated seedlings, a three hour WD resulted in a slight increase, and a six hour WD resulted in a decrease in the activity of the examined enzyme. In the Se pre-treated roots, a higher activity was found after a three hour WD at 1 and 10 μM of Se concentrations and after a six hour WD at 5 and 10 μM of Se, compared to the control without a WD. The highest activity of APX was observed in the roots pre-treated with 10 µM of Se after 3rd and 6th hour of the WD. It was higher by, on average, 104.3%, compared with the activity in roots only pre-treated with Se but without a WD.

#### 2.2.2. POX Activity

**Figure 5:**
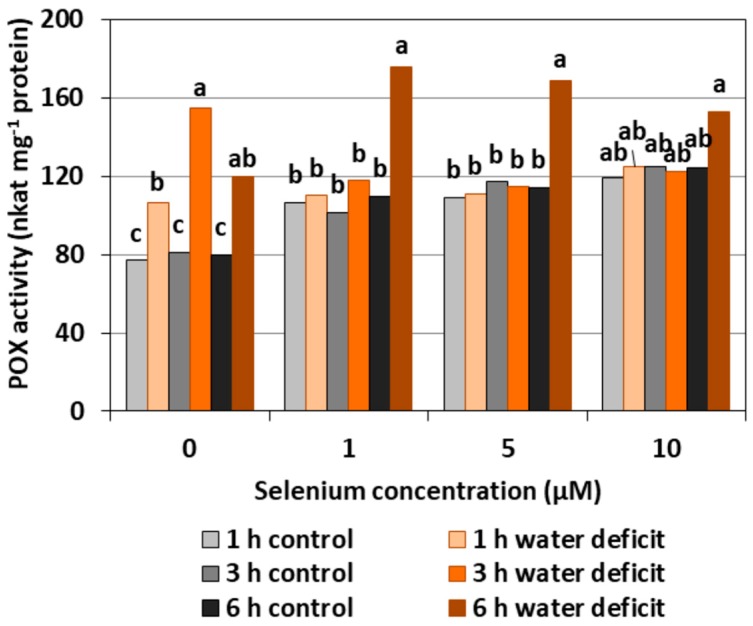
The peroxidase (POX) activity in the roots of cucumber seedlings pre-treated with Se and subjected to a WD. Bars marked with the same letters do not differ significantly at *p* < 0.05.

The pre-treatment with Se of the roots of cucumber seedlings caused a significant increase in POX activity at a similar level regardless of the applied concentration of Se by, on average, 43.5% (Figure 5). A WD in Se untreated roots also created an increase in the activity of the examined enzyme. The highest POX activity was recorded in the roots of cucumber seedlings pre-treated with Se in all concentrations and then subjected to a WD for six hours. At this time, the synergistic effect of the Se treatment and a WD occurred.

#### 2.2.3. CAT Activity

**Figure 6:**
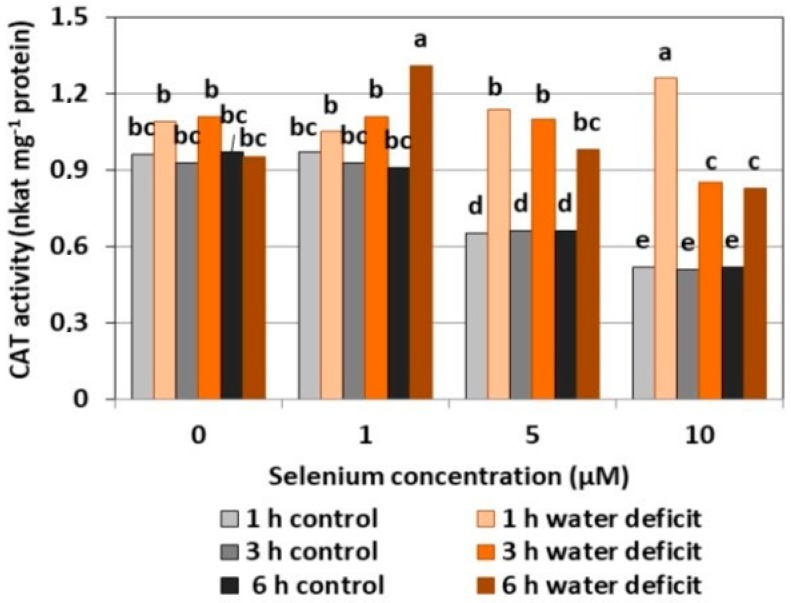
The catalase (CAT) activity in the roots of cucumber seedlings pre-treated with Se and subjected to a WD. Bars marked with the same letters do not differ significantly at *p* < 0.05.

The selenium pre-treatment at a concentration of 1 μM did not cause significant changes in the CAT activity as compared to the control without Se (Figure 6). The treatment of Se at 5 and 10 μM resulted in a decreased activity of the examined enzyme compared to the activity in the Se untreated roots, respectively, by 31.2% and 45.4%. A significant increase in CAT activity was found in the roots pre-treated with 1μM of Se and a six hour WD and with 10 μM of Se and a one hour WD, which was, on average, more than 1.5 times higher than the activity of CAT in Se-treated roots without a WD. T In these cases, the effect of the interaction between Se and a WD on the increase of CAT activity in the roots of the cucumber seedlings was noticeable.

#### 2.2.4. SOD Activity

**Figure 7:**
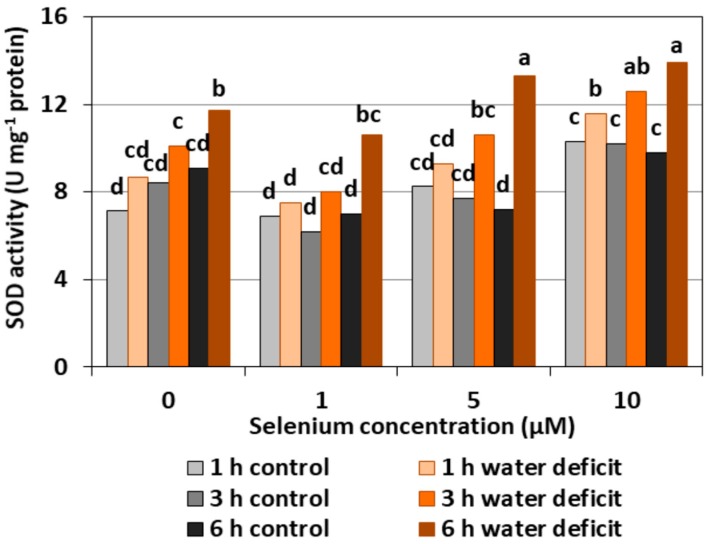
The superoxide dismutase (SOD) activity in the roots of cucumber seedlings pre-treated with Se and subjected to a WD. Bars marked with the same letters do not differ significantly at *p* < 0.05.

The pre-treatment of cucumber seedlings with Se did not significantly affect the changes of SOD activity in the roots. The exception was the roots treated with 10 μM of Se, in which there was an upward trend in the activity of the examined enzyme—not always statistically proven—compared to the controls without Se and other variants with Se treatment (Figure 7). The highest SOD activity of, on average, 63.4% was observed in the roots of cucumber seedlings treated with Se at 5 and 10 µM concentrations and subjected to a six hour WD in comparison to Se-treated roots without a WD.

### 2.3. Lipid Peroxidation

**Figure 8:**
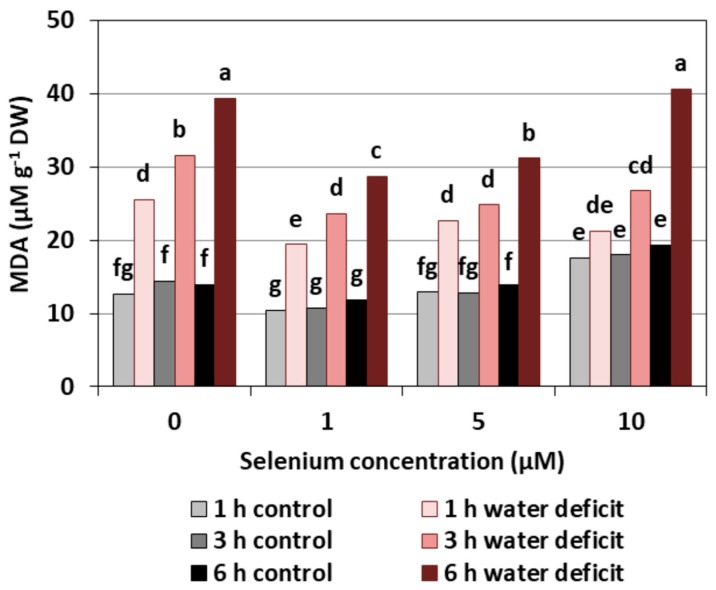
The effect of Se on malondialdehyde (MDA) concentrations in the roots of cucumber seedlings pre-treated with Se and subjected to a WD. Bars marked with the same letters do not differ significantly at *p* < 0.05.

Se treatment created a decrease of the MDA level, a product of lipid peroxidation, only in the roots of cucumber seedlings pre-treated with 1 µM of Se. However, the effect of Se on the increase of the MDA level was observed only in the roots of cucumber seedlings treated with Se in the highest concentration (Figure 8). It was, on average, 33.5% higher than roots not treated with Se. The introduction of a WD clearly increased the content of MDA, and the longer a WD lasted, the higher the MDA level was. The pre-treatment of cucumber seedlings with Se at concentrations of 1 and 5 μM significantly reduced the effect of a WD on the production of MDA in the roots by, on average, 22.1% compared to the control without Se.

### 2.4. Impact of Se on the Damage of Cell Membranes Induced by a WD

**Figure 9:**
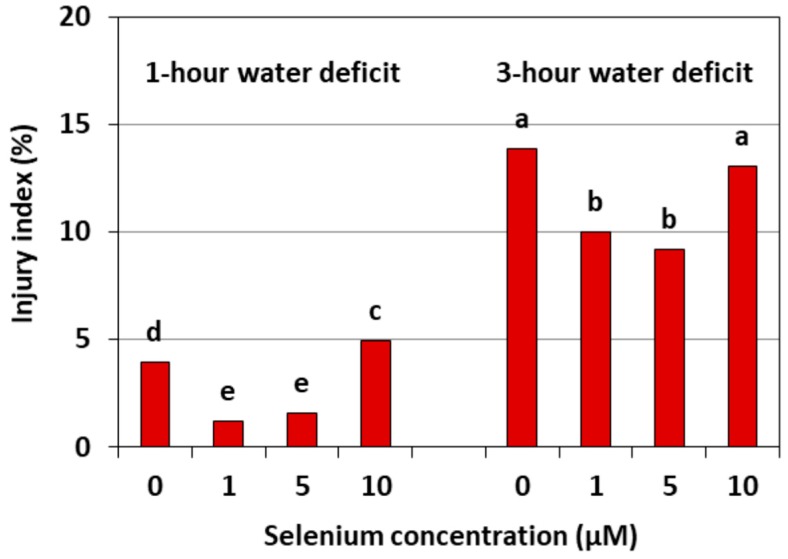
The damage of cell membranes in the roots of cucumber seedlings pre-treated with Se and subjected to a WD. Bars marked with the same letters do not differ significantly at *p* < 0.05.

Figure 9 shows the damage to the cell membranes of cucumber seedling roots caused by a WD. This damage is expressed by an injury index relative to the control without a WD. The strongest damage to the cell membranes was found in the roots of cucumber seedlings, both untreated with Se and pre-treated with 10 μM of Se, after three hours of a WD. The injury index in these roots was, on average, around 14%. However, the index in the roots of the cucumber seedlings pre-treated with 1 and 5 μM of Se and subjected to a WD was smaller than in the roots without Se and was, on average, 65.2% after one hour and 30.8% after three hours of a WD.

### 2.5. Water Content

**Figure 10:**
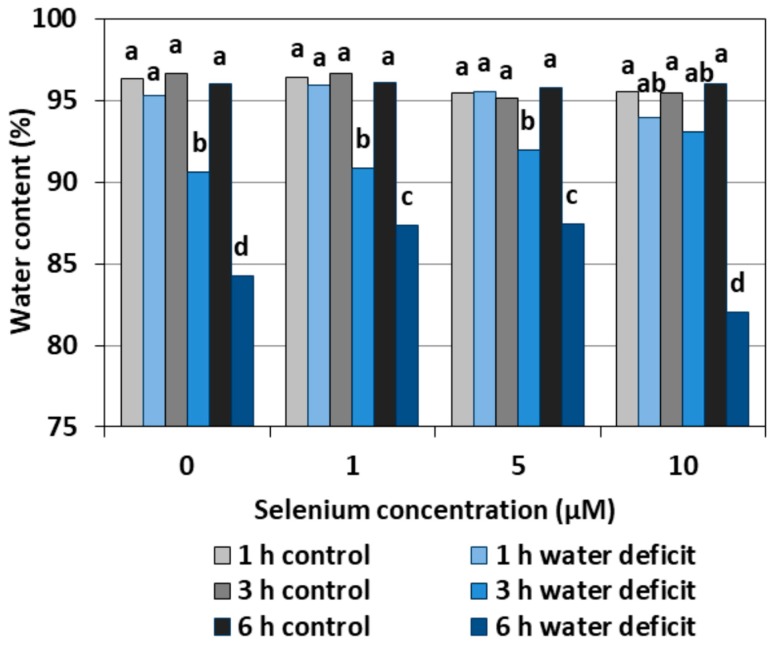
The water content in roots of cucumber seedlings pre-treated with Se and subjected to a WD. Bars marked with the same letters do not differ significantly at *p* < 0.05.

A WD caused a significant reduction in the water content in the roots of cucumber seedlings both pre-treated and untreated with Se, in relation to the roots not subjected to a WD (Figure 10). The lowest water content was found in the roots treated with a WD for six hours and untreated with Se or pre-treated with 10 µM of Se. The beneficial effect of Se was observed only in the roots of seedlings pre-treated with Se at 1 μM and 5 μM concentrations after six hours of stress, in which the water content was significantly higher than in the control without Se.

## 3. Discussion

The results of our previous work showed that the addition of sodium selenite to a nutrient solution resulted in the accumulation of Se in cucumber seedlings [13]. The accumulation of Se in seedling roots was proportional to Se concentrations. The content of Se in the roots of the control seedlings was 444.12 μg kg^−1^ dry weight. In the roots growing in the nutrient solution with the addition of sodium selenite at the concentrations of 1, 5 and 10 μM, the content of Se was, respectively, 524.06, 604.00, and 759.4 μg kg^−1^ dry weight.

There is more and more evidence in the literature indicating that the nutrition of plants with Se not only increases their biological value [14,15] but can also mitigate the effects of various stress factors [9,16]. The interest in issues concerning the role of Se in alleviating the negative effects of environmental stress, mainly abiotic, is still growing [17]. It has been shown that some species of plants in the presence of Se were characterized by an increased tolerance to stress factors such as heavy metals, drought, extremal temperatures, and ultraviolet radiation [9].

Depending on the form in which Se occurs, its concentration and accumulation in tissues as well plant species, Se in low concentrations may play an important role in the metabolism of the antioxidant system; however, in higher concentrations, Se may cause a pro-oxidative effect, which indicates the dual nature of this element [18]. Our studies carried out on the roots of cucumber seedlings showed that Se in the form of selenite in the highest concentration (10 μM) increased the generation of O_2_^•−^, H_2_O_2_, and ^•^OH (Figure 1, Figure 2 and Figure 3). However, at lower concentrations of Se (1 and 5 μM), this effect was not observed. Lower O_2_^•−^ levels were found in the roots of seedlings pre-treated with 1 and 5 μM of Se and subjected to a WD for three and six hours compared to the roots of seedlings untreated with Se (Figure 1). At the same time, the statistically significant weaker generation of H_2_O_2_ in roots of seedlings pre-treated with 1 μM of Se and subjected to a six hour WD was also demonstrated, as compared to the ones subjected only to a WD (Figure 2). In addition, there was generally a noticeably lower generation of ^•^OH in the roots of seedlings pre-treated with 1 and 5 μM of Se and with a WD compared to the action of a WD alone (Figure 3).

Research by other authors confirms that Se can cause an increased ROS generation in plants [19,20]. Chen et al. [21] observed that Se in the form of selenite caused a generally increased generation of ROS, including O_2_^•−^ in the roots of *Brassica rapa*. They used a very wide range of concentrations of sodium selenite, i.e., from 0.03 to 0.46 mM, and showed that the higher the concentration of Se and the longer its action, the stronger the generation of ROS. Increasing the level of H_2_O_2_ in lettuce with the increase in the concentration of Se was shown by Ríos et al. [22]. In addition, these authors observed that the effect of Se on the generation and scavenging of H_2_O_2_ was dependent, to a large extent, on the form of this element. It was found that the generation of this molecule was stronger during the interaction of Se in the form of selenite than selenate. It was shown that the use of 20 μM of selenite already caused a significant difference in the generation of H_2_O_2_ compared to the control, while a similar effect after treatment with selenate was obtained only at a concentration of 120 μM. Selenium with a concentration higher than 10 μM also caused an increase in lipid peroxidation. In the roots of seedlings growing in the presence of lead in toxic concentrations, the addition of 1.5 μM of Se increased the cell viability, glutathione content, and total content of thiol groups, while, in a higher 6 μM concentration, it significantly increased O_2_^•−^, decreased cell viability, and decreased total thiol content [20]. Thus, the occurrence of an antioxidant or pro-oxidative effect depends on the concentration of Se used.

A small addition of Se to the substrate reduces the generation of ROS, especially O_2_^•−^ and H_2_O_2_, in plant cells undergoing various stress factors. Cartes et al. [23] showed that Se in the form of selenite alleviated the oxidative stress induced by the presence of aluminium in the roots of ryegrass, mainly by increasing the spontaneous O_2_^•−^ to H_2_O_2_ dismutation. Mroczek-Zdyrska and Wójcik [20] found that 1.5 μM of sodium selenite reduced the O_2_^•−^ level in the roots of broad bean seedlings (*Vicia faba*) growing in the environment contaminated with cadmium.

Wang [24] showed that 5 μM of sodium selenate reduced the accumulation of H_2_O_2_ in the leaves of *Trifolium repens* seedlings growing in WD conditions. A reduction in the generation of ROS under the influence of Se has also been demonstrated for other species under stress conditions, including in sorghum (*Sorghum bicolor*) seedlings exposed to high temperatures, with lower levels of O_2_^•−^ and H_2_O_2_ in the presence of Se [25]. In addition, in wheat (*Triticum aestivum*), Se reduced the level of O_2_^•−^ under drought stress [26].

An appropriate concentration of Se may affect the level of ROS, including O_2_^•−^, which takes place through three processes: The spontaneous O_2_^•−^ dismutation to H_2_O_2_ (in a non-catalysed by an SOD reaction), the direct removal of O_2_^•−^ and H_2_O_2_ by Se compounds, and the regulation of antioxidant enzyme activity [18,23]. It is worth noting that ROS generation under stress conditions can exert an effect that is not only damaging, because both H_2_O_2_ and O_2_^•−^ assign a signalling role in the induction of defence and acclimatization processes to stress factors and unfavourable environmental conditions [10].

In general, a higher activity of antioxidant enzymes such as APX, POX, CAT, and SOD was found in the roots of cucumber seedlings pre-treated with Se and subjected to a WD in comparison to the action of the stress factor itself. The increase in the activity of these enzymes was dependent on the concentration of Se and the duration of the WD (Figure 4, Figure 5, Figure 6 and Figure 7). The decrease in CAT activity recorded in our work using 5 and 10 μM of Se compared to the control without Se (Figure 6) was an interesting result, because the level of H_2_O_2_ was generally close to the control (Figure 2). Therefore, on the basis of the obtained results, it can be concluded that, among the designated antioxidant enzymes in the roots of cucumber seedlings, POX, but not CAT, is responsible for maintaining a low level of H_2_O_2_.

In our work, we also observed ROS generation in the roots of cucumber seedlings subjected to a WD. An increased ROS generation and changes in the activity of antioxidant enzymes are a frequently observed form of plant response to a WD. Zhang et al. [27] showed that a WD limited the root growth of cotton seedlings in the drought-sensitive variety, stimulated the generation of H_2_O_2_, and increased the content of MDA. However, they did not find any changes in the activity of CAT, APX, and SOD in this variety. In the drought-resistant variety, the stimulation of root growth was found, yet there were no changes in the MDA level, nor an increased activity of CAT, APX, and SOD. Fan et al. [3] showed a correlation between a WD, oxidative stress, and lipid peroxidation. These authors also observed an increase in the activity of antioxidant enzymes such as SOD, POX, CAT, and APX depending on the duration and intensity of a WD. They also found that an increased ROS production during severe drought stress is not always accompanied by an increase in antioxidant enzymes. Hasanuzzaman and Fujita [28] showed that the use of Se contributed to a significant reduction of oxidative stress in rape seedlings by an increase in the activity of many enzymes of the antioxidant system, including CAT and APX, and significantly reduced H_2_O_2_ and MDA levels. Wang [24] also found an increase in the activities of antioxidant enzymes (APX, SOD, CAT, and glutathione reductase) in the leaves of *Trifolium repens* seedlings subjected to a WD. The activities of these enzymes, except CAT, were even higher in seedlings treated with Se and subjected to a WD. In our study, the activity of SOD in WD conditions generally remained at a level similar to the control without a WD (Figure 7). Se pre-treatment, especially at higher concentrations and with prolonged WD action, significantly increased the activity of this enzyme. Literature data indicate that SOD activity varies in plants growing in the presence of Se and subjected to stress factors. The analysis of the work of many authors draws attention to three main factors that can affect the activity of this enzyme. The first factor is the intensity of oxidative stress. At low stress levels, the production and removal of ROS is mainly controlled by glutathione peroxidase and by the spontaneous reduction of O_2_^•−^ which limits the function of SOD [18]. However, along with the intensity of stress, the generation of ROS increases, and, at the same time, there is a need to include another enzyme of the antioxidant system. These authors observed a low SOD activity in young ryegrass seedlings and an increased SOD activity in aging plants. A similar relationship was demonstrated in lettuce by Xue et al. [29]. Another factor, apart from the level of stress, which influenced the activity of SOD, was the concentration of Se. Cartes et al. [23] showed that 2 μM of Se in the form of selenite significantly reduced the activity of SOD in seedlings of rye treated with aluminium in a toxic concentration, while 10 μM of Se contributed to an increased activity of this enzyme. Ríos et al. [22] also showed an increased SOD activity with an increasing Se dose. A significant increase in the activity of this enzyme compared to the control was observed already at the concentration of 20 μM of selenite, although the highest SOD activity was noted after the application of 40 μM. An increased SOD activity may suggest increased O_2_^•−^ generation due to the toxicity of Se. The third factor determining SOD activity may be the varied concentration of SOD cofactors, i.e., iron, manganese, copper, and zinc. It was found that the treatment of pea seedlings with 10 and 20 µM of Se in the form of selenite decreased the accumulation of these micronutrients [30].

The results of other authors indicate that Se at lower concentrations acts as an antioxidant reduces the degree of lipid peroxidation, while Se at higher concentrations enhances lipid peroxidation [29,31]. Hartikainen et al. [18] showed that Se in the highest doses (10–30 mg kg^−1^) had a highly toxic effect, and it was proven that this element may act as a pro-oxidant which increases lipid peroxidation tenfold in ryegrass. In contrast, the use of Se at a lower dose, i.e., 1.0 mg kg^−1^, stimulated the antioxidant capacity in this species by manifesting a reduction in the degree of lipid peroxidation. Though Sharma et al. [32] showed that the degree of lipid peroxidation in rape seedlings was higher compared to the control in the case of selenate treatment (4 mg Se kg^−1^ of soil), a similar concentration of selenite caused a reduction in lipid peroxidation.

The results obtained in our work showed that, in the roots of cucumber seedlings pre-treated with 10 μM of Se, a significant increase in MDA level was observed (Figure 8). At the highest Se concentration used, an increased generation of O_2_^•−^ was also observed (Figure 1). This is probably related to the activation of the antioxidant system in the roots of cucumber seedlings. Simultaneously there was also an increase in POX activity in the roots of cucumber seedlings pre-treated with 5 and 10 μM of Se (Figure 5) and an increase in SOD activity at the highest concentration of Se in relation to the control without Se (Figure 7). Numerous studies have shown that Se antioxidant activity in higher plants is often associated with an increase in glutathione POX activity and a reduction in lipid peroxidation [18].

Our work demonstrates the lowest level of lipid peroxidation product—MDA—and the smallest damage to plasma membranes caused in the roots of cucumber seedlings pre-treated with 1 and 5 μM of Se and subjected to a WD (Figure 8 and Figure 9). Hawrylak-Nowak [33] showed that the introduction of 5 and 10 μM of Se to a nutrient solution in the form of selenite decreased the lipid peroxidation and cell membrane damage in cucumber seedlings treated with NaCl at a stressful concentration. These results indicate the antioxidant activity of this element and may indicate its beneficial effect on the integrity of cell membranes under stress conditions.

The results obtained in our work confirm that Se can play a role in the mechanism of cucumber seedling root tolerance to a WD, which is associated with increased antioxidant capacity. The latter resulted in a small but statistically proven increase in the water content of the roots of cucumber seedlings treated with 1 and 5 μM of Se in the six hours of a water deficit (Figure 10). Further research is needed in the long term to show what the effects of the changes caused by Se will be.

## 4. Material and Methods

### 4.1. Preparation of Plant Material

Cucumber seeds (Cucumis sativus L. cultivar Dar) were soaked in redistilled water for 30 min and then surface sterilized by immersion in 0.5% sodium hypochlorite (NaClO) for 5 min. This was followed by 5 rinses with distilled water. After 20 h, the germinated seeds were placed on vertically positioned plates with moist filter paper in a thermostat for 6 days. The homogenous and best-developed seedlings were then transferred into glass chambers with a capacity of 1.9 dm3 with a 1/5 strength aerated Hoagland No 1 nutrient solution for the next 4 days. The experiment was carried out in a controlled-environment growth chamber with the following conditions: A 12 h day/night period with a light intensity of 140 W m^–2^ (Philips lamps) with 27/23 °C day/night temperatures and a relative air humidity of about 50–55%.

### 4.2. Selenium Treatment

**Figure 11:**
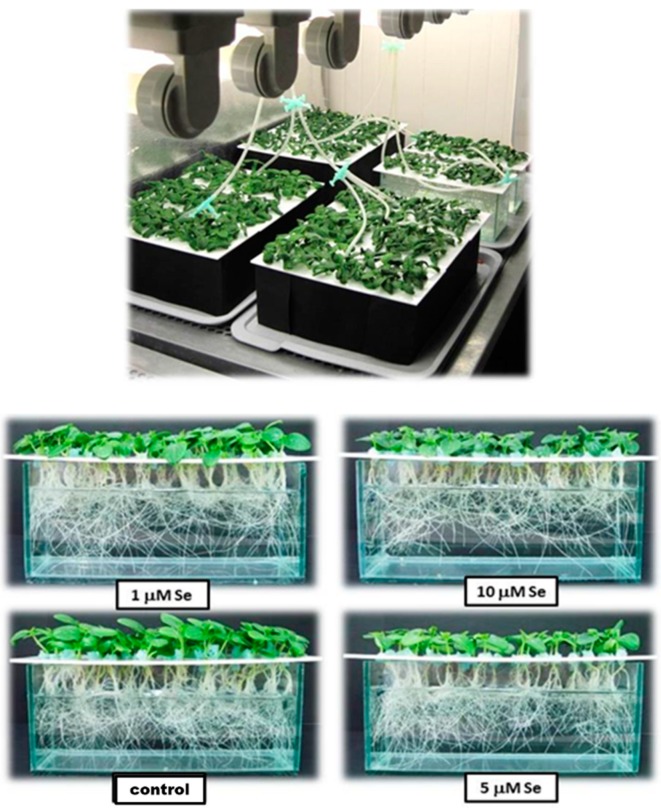
Seedlings of cucumber pre-treated with Se in the growth chamber before introducing the WD.

Selenium was added to the nutrient solution as sodium selenite (Na_2_SeO_3_·5H_2_O) at concentrations of 1, 5, and 10 µM. Each treatment had three replicates of fifty seedlings. Cucumber seedlings were grown in nutrient solution with sodium selenite for 96 h (Figure 11).

After 96 h of growth in the nutrient solution with sodium selenite at various concentrations, the seedlings were subjected to a WD for 1, 3 and 6 h, and then root samples were taken for the determination of ROS (O_2_^•−^, H_2_O_2_, ^•^OH), antioxidant enzymes (APX, POX, CAT, SOD), MDA, cell membrane damage, and water content.

### 4.3. WD Introduction

After 96 h of Se treatments, the cucumber seedlings were subjected to a WD by surface dehydration. The roots were removed from the nutrient solution, washed 3 times with distilled water, and placed in empty glass chambers. A similar way of introducing a WD was used in cucumber studies by Kubiś et al. [34].

### 4.4. Determination of ROS

All measurements of ROS were performed in three independent experimental repetitions and were carried out spectrophotometrically (PerkinElmer Lambda 15 UV-Vis spectrophotometer).

#### 4.4.1. Superoxide Anion (O_2_^•−^)

O_2_^•−^ was determined on the basis of its ability to reduce nitroblue tetrazolium (NBT, Lab Empire) [35]. Four roots were collected for the assays, and then, without damaging them, they were placed in 3.0 cm^3^ of a 0.01 M potassium phosphate buffer at pH 7.8 with the addition of 0.05% NBT and 0.5 mM NaN_3_. The reference sample was an incubation mixture without roots. The samples were incubated for one hour at room temperature and shook every 15 min. For measurement, 2.0 mL of the incubation mixture was collected and heated in a water bath at 80 °C for 15 min. After cooling in ice to room temperature, the absorbance of the solution was measured at 580 nm. O_2_^•−^ content was expressed in absorbance units per 1 g dry weight of roots.

#### 4.4.2. Hydrogen Peroxide (H_2_O_2_)

H_2_O_2_ content was determined according to the method of Becana et al. [36]. Four roots of cucumber seedlings were homogenized in a cooled mortar with 3 mL of 5% trichloroacetic acid (TCA, Serva) and 0.1 g of active carbon. The homogenates were filtered through a membrane layer—miracloth (Biofuge fresco Heraeus, Kendro Laboratory Products, USA)—and centrifuged at 12,000 × g for 30 min at 4 °C. After centrifugation, the pellet was discarded, and the supernatant was used to determine H_2_O_2_ content. The reaction mixture contained 2.0 mL of a 0.1 mM potassium phosphate buffer at pH 8.4, 60 μL of the analysed extract, and 1 mL of the titanium reagent. The titanium reagent was prepared by mixing it in a 1: 1 (v/v) ratio 0.6 mM solution of 4-(2-pyridylazo) resorcinol and 0.6 mM potassium titanate oxalate (Sigma-Aldrich). The reference sample was 5% TCA. The absorbance of the complex formed was measured at a wavelength of 508 nm, and the H_2_O_2_ content in the tissue was read from the standard curve. The H_2_O_2_ content was expressed in nM·g^−1^ dry weight of roots. The assays were performed in 3 independent experimental repetition.

#### 4.4.3. Hydroxyl Radical (^•^OH)

The content of ^•^OH was determined by the method of von Tiedemann [37], which consists of the measurement of the pink complex resulting from combining the product of deoxyribose degradation by ^•^OH with thiobarbituric acid (TBA). Four roots were taken and immersed in 2.4 mL of 1 mM deoxyribose (Sigma-Aldrich). The samples were incubated for 1 h in the dark, at room temperature. For measurements, 0.5 mL of the incubation mixture was collected, and 0.5 mL of 2.8% TCA and 0.5 mL TBA in 0.05 M NaOH were added and boiled in a water bath at 100 °C for 10 min. After cooling on ice to room temperature, the absorbance of the solution was measured at 540 nm. The ^•^OH content was expressed in absorbance units per 1 g dry matter.

### 4.5. Extraction of Roots for the Determination of Antioxidant Enzyme Activities, Protein Content and Level of Lipid Peroxidation

In order to achieve the unified conditions of extraction, 5 roots were weighed and homogenized in cooled mortars with 4.0 mL of a 0.1 mM potassium phosphate buffer at pH 7.0 (with 30 mg of Polyclar AT). The extract was centrifuged for 30 min at 15,000× *g* at 4 °C. The obtained supernatant was used to determine the activities of APX, CAT, and POX, as well as the protein content and MDA level.

Another extraction method was used to determine the SOD activity. Five roots were weighed and homogenized in cold mortars with 3 mL of a 0.05 mM sodium phosphate buffer at pH 7.0, with 1% PVP, 1 mM EDTA-Na, and 0.5% NaCl. The homogenate was centrifuged for 30 min at 15,000× *g* at 4 °C.

### 4.6. Determination of Antioxidant Enzyme Activities

The assays were performed in three independent experimental repetitions. Measurements of enzyme activities were carried out spectrophotometrically (UV-VIS Jasco spectrophotometer with V-530 software for Windows).

#### 4.6.1. APX Assay

APX activity (EC 1.11.1.11) was determined based on the Nakano–Assada [38] method. The reaction mixture contained 2.3 mL of a 0.1 mM potassium phosphate buffer at pH 7.0, to which 0.2 cm^3^ of extract and 0.2 mL of 5 mM ascorbic acid were added. The reaction mixture was placed in a quartz cuvette, and 0.3 mL of H_2_O_2_ was added. The decrease in the absorbance of the sample caused by the oxidation of ascorbate was measured for 2 min at the wavelength λ = 290 nm. APX activity was calculated on the basis of the molar absorption coefficient for L-ascorbate, which is 2.8 mM^−1^ cm^−1^, [39] and expressed in nkat mg^−1^ protein.

#### 4.6.2. POX Assay

POX activity (EC 1.11.1.7) was determined according to the method of Hammerschmidt et al. [40]. The reaction mixture contained 0.5 mL 20-fold diluted with the extract buffer, to which 0.5 mL of guaiacol at 3.4 mM was added. The reaction was initiated by adding 0.5 mL of 3% H_2_O_2_ (Sigma-Aldrich). The absorbance increase caused by the oxidation of the substrate was measured at wavelength λ = 480 nm, for 2 min. POX activity was calculated on the basis of the molar absorption coefficient for tetraguaiacol of 26.6 mM^−1^ cm^−1^ [41] and expressed in nkat∙mg^−1^ protein.

#### 4.6.3. CAT Assay

CAT activity (EC 1.11.1.6) was determined according to the method of Dhindsa et al. [42]. The reaction mixture (3 mL) contained a 0.1 mM potassium phosphate buffer at pH 7.0 and 0.1 mL of enzyme extract. The reaction was initiated by adding 3% H_2_O_2_. The absorbance loss at wavelength λ = 240 nm was measured for 2 min. The activity of CAT was expressed in nkat mg^−1^ protein, assuming a molar absorption coefficient for H_2_O_2_ 39.4 μM^−1^ cm^−1^ [43].

#### 4.6.4. SOD Assay

SOD activity (EC 1.15.1.1) was determined according to the method of Beauchamp and Fridovich [44], using the ability of this enzyme to inhibit the photochemical reduction of nitroblue tetrazolium (NBT). The reaction mixture (3 mL) contained a 0.05 mM phosphate buffer at pH 7.8, 97 mM methionine, 2 mM NBT, enzymatic extract, and 120 μM riboflavin. The reaction was initiated by adding riboflavin. Cuvettes with the reaction mixture were exposed to UV light for 15 min. Meanwhile, a reference sample (without the enzyme extract) was incubated in parallel. The measurement was carried out at the wavelength λ = 560 nm. The result was converted into the number of enzymatic units of enzymatic activity (U) per mg of protein, with 1 U being the amount of SOD that inhibited the NBT reduction by about 50%. SOD activity was expressed in U mg^−1^ protein.

#### 4.6.5. Protein Content

The protein content was measured spectrophotometrically according to the Bradford method [45]. Up to 25 μL of extract was added to 1.975 mL of Coomassie Brilliant Blue. Absorbance was measured after 10 min at a wavelength λ = 595 nm (Jasco UV-Vis spectrophotometer with software V-530 for Windows). The protein content was read from the standard curve of bovine albumin and was used to express the activities of antioxidant enzymes.

### 4.7. Lipid Peroxidation

The degree of lipid peroxidation was determined according to the method of Heath and Packer [46], which involves the testing of the level of MDA as a lipid peroxidation product by means of a coloured reaction with TBA. As a result, a colourful product was formed whose concentration in the sample can be determined by a spectrophotometric method. The tubes containing 500 μL of the supernatant were filled with 2.0 mL of 0.5% TBA in 20% TCA and placed in a boiling water bath for 30 min. After this time, the tubes were quickly cooled and then centrifuged for 10 min at 12,000× *g*. The absorbance of the supernatant was measured at wavelength λ = 532 nm (A_532_) and 600 nm (A_600_). The result was corrected by subtracting from A532 the A600 value resulting from the reaction of non-specific products with TBA (UV-Vis Jasco spectrophotometer with V-530 software for Windows). The concentration of MDA was calculated from the molar absorption coefficient (155 mM^−1^·cm^−1^) according to the formula:

$$C=\frac{A_{532}–A_{600}}{155}\mathrm{mM}$$

### 4.8. Damage of Cell Membranes

Damage to cell membranes was determined according to the method described by Kacperska [47]. The roots of five cucumber seedlings were placed in 30 mL beakers and rinsed three times with deionized water. Samples of the control combination without Se and a WD were flooded with 10 mL of deionized water. The roots subjected to a WD were dehydrated beforehand for 1 and 3 h, and then were flooded with 10 mL of deionized water. After 1 or 3 h of root incubation in deionized water, the measurements of the electrolytic conductivity of the solutions were carried out (conductivity meter Elmetron CC-411). Then the samples were covered and boiled for 5 min, and, after cooling, the electric conductivity of the solution was measured again. The leakage of electrolytes from roots was the criterion of membrane damage. The degree of damage was calculated according to the formula:

$$I_{\mathrm{in}}=\frac{C_{\mathrm{WD}}-C_{c}}{C_{t}-C_{c}}\times 100\%$$

where I_in_ is the injury index, C_WD_ is the conductivity of solution from the roots subjected to a WD, Cc is the conductivity of solution from the control roots, and C_t_ is the conductivity of the solution after boiling.

### 4.9. Water Content

The freshly cut roots of the cucumber seedlings were weighed, placed in weighing bottles, and dried for 72 h at 70 °C. Then, the samples were again weighed to determine the dry matter. The water content in the roots was calculated according to the formula [48]:

$${\%H}_{2}O=\frac{\mathrm{fresh}\;\mathrm{weight}-\mathrm{dry}\;\mathrm{weigh}}{\mathrm{fresh}\;\mathrm{weight}}\times 100\%$$

### 4.10. Statistical Analysis

The obtained results were subjected to statistical analysis by the Statistica 9 program (StatSoft Inc., USA) and SigmaPlot ver. 11.0 (Systat Software Inc., San Jose, CA, USA). The evaluation of the influence of the time, the WD, and the Se concentration on the studied parameters was made on the basis of three-way ANOVA. Significant differences were detected by the Tukey test at the significance level *p* < 0.05. Before applying the analysis of variance, the empirical data expressed as a percentage (p) was transformed according to the Bliss formula:

$$z=a\;\sin\sqrt{p}$$

## 5. Conclusions

Se at concentrations of 1 and 5 μM increased tolerance of cucumber seedlings to oxidative stress caused by a WD by increasing the enzymes of the antioxidant system, mainly APX and CAT, as well as by limiting the damage of plasma membranes as a result of suppression of lipid peroxidation. Se at a concentration of 10 μM was toxic to the cucumber roots, intensifying the generation of ROS, which resulted in lipid peroxidation and damage to the plasma membranes.
